# Epidemiological investigation of Shuni virus infections in hospitals in two provinces of South Africa (2019–2021) using molecular and serological surveillance

**DOI:** 10.1371/journal.pntd.0014738

**Published:** 2026-09-21

**Authors:** Matshepo Elizabeth Rakaki, Adriano Mendes, Paul Wichgers Schreur, Martin H. Groschup, Markus Keller, Nicolette Marie Du Plessis, Maryke De Villier, Theunis Avenant, Marietjie Venter

**Affiliations:** 1 Centre for Emerging Arbo and Respiratory Virus Research (CEARV), Department of Medical Virology, Faculty of Health Sciences, University of Pretoria, Pretoria, South Africa; 2 Wageningen Bioveterinary Research, Wageningen, The Netherlands; 3 Institute of Novel and Emerging Infectious Diseases, Friedrich-Loeffler-Institut, Greifswald, Germany; 4 Department of Paediatrics, Kalafong Provincial Tertiary Hospital, University of Pretoria, Pretoria, South Africa; 5 Emerging Viral Threats, One Health Surveillance and Vaccines (EViTOH) South African Research Chair (SARChi), Infectious Disease and Oncology Research Institute (IDORI), University of the Witwatersrand, Johannesburg, South Africa; Medizinische Universitat Wien, AUSTRIA

## Abstract

Shuni Virus (SHUV) is a reemerging zoonotic orthobunyavirus in the Peribunyaviridae family associated with neurological infections and birth defects in humans and animals in Africa and has emerged in the Middle East in the past 10 years. Limited epidemiological data exist in humans, partially due to a lack of clinical awareness and availability of diagnostic assays to determine the incidence of clinical cases and seroprevalence in the population. The goal of this study was to establish serologic and molecular diagnostic assays for SHUV for hospital-based surveillance and to investigate the clinical epidemiology in humans in South Africa. The incidence of SHUV infections was investigated in patients with acute fever of unknown cause with or without neurological signs (AFDUC/N) during the arbovirus season (January-June, 2019–2021). IgM and IgG ELISAs for SHUV were established using baculovirus-expressed glycoproteins and validated against virus-neutralization tests (VNT) positive sera. Orthobunyavirus quantitative RT-PCR (RT-qPCR) and IgM ELISA were used to identify acute infections, and IgG ELISA to define the seroprevalence in patients and healthy control groups in hospitals in Gauteng and Mpumalanga provinces in South Africa. In total, 3/349 (0.84%) AFDUC/N cases tested positive by RT-qPCR and confirmed as SHUV by sequencing. In total, 22/225 (9.7%) AFDUC/N patients had SHUV neutralising antibodies (VNT), of which 11/22 (50.00%) were IgM positive, with an IgM seropositivity of 4.89% (11/225 (4.89%)). Of the RT-PCR and double IgM + VNT+ patients, 60% presented with seizures and 40% with meningitis, of which 36.36% were children and 63.63% were adults, respectively. In total, 9/112 (8%) of the healthy control group tested positive on the IgG ELISA assay. These findings suggest that SHUV is a missed cause of acute neurological infections in hospitalized children and adults in South Africa and should be investigated in humans in Africa and other regions.

## Author summary

SHUV is a mosquito and biting midge-transmitted zoonotic virus that causes neurological disease and birth defects in humans and animals in Africa and the Middle East, yet it remains severely underdiagnosed due to limited clinical awareness and the absence of commercially available diagnostic tools. In this study, we developed and validated IgM and IgG ELISAs for SHUV in humans and applied them alongside molecular testing to investigate SHUV infections in hospitalized patients presenting with unexplained fever and/or neurological signs in Gauteng and Mpumalanga provinces of South Africa between 2019 and 2021. We found that combining molecular and serological testing identified SHUV in approximately 6% of cases during the arbovirus season, with patients presenting with febrile convulsions, seizures, and meningitis affecting both children and adults. Importantly, an 8% seroprevalence in healthy controls suggests broader exposure than previously recognized. These findings highlight SHUV as a missed but clinically important cause of neurological illness in South Africa, and the need for expanded surveillance across Africa and other regions where SHUV may be circulating undetected.

## Introduction

Shuni virus (SHUV) is a zoonotic orthobunyavirus in the *Peribunyaviridae* family. It has been associated with neurological infections and birth defects in humans and animals in South Africa [1–4] and recently emerged in Israel, where it caused disease outbreaks in cattle and sheep [5–7]. The *Orthobunyavirus* genus comprises a large group of > 189 viruses and 18 serogroups, including several viruses of human and veterinary health importance. Orthobunyaviruses are transmitted by mosquitoes and Culicoides biting midges [8–13]. Several orthobunyaviruses emerged in new regions in recent years, including Oropouche virus (OROV) in humans in South America, the Caribbean, and Europe [14–16] and Schmallenberg virus (SBV) in livestock across Europe [17–22]. Schmallenberg virus likely originated in Africa and has since been detected across the continent, emphasizing the importance of arbovirus surveillance in Africa [17–19,22,23]. Orthobunyaviruses that have an impact on human and animal health in Africa include SHUV, Ngari virus (NRIV), and Bunyamwera virus (BUNV) [1–4,24–29].

The first case of SHUV in a human was identified in 1966 from the blood of a febrile child during a surveillance program at the General Outpatients Clinic at the University College Hospital (UCHGOP) in Nigeria [30]. The first reports of SHUV in South Africa were isolated from Culex theileri mosquitoes, cattle, a goat, and horses in the 1970s [31,32]. SHUV was detected again in 2009 in horses and subsequently described as a missed cause of neurological infections and birth defects in horses [2–4], wildlife, livestock [3], and humans [1] in South Africa and since 2012, in birth defects and neurological infections in cattle and sheep in Israel [5,6]. SHUV has been identified in various Culex (Cx) and Aedes (Ae); Anopheles (An) and Mansonia (Ma) mosquito vectors, including An. pharoensis, Cx. theileri, Cx. annulioris, Ma. Africana, Ma. uniformis, Aedes mcintoshi, Ae. subargenteus [12,32]. In addition, it has also been identified in biting midge vectors such as Culicoides nubeculosus, C. sonorensis, and Culicoides spp [11,13,33].

The first human SHUV cases were identified in South Africa in cerebrospinal fluid (CSF) samples of patients hospitalized in Gauteng province during the arbovirus season in January–May 2017 using molecular testing [1]. In this pilot study, 7/130 (5.4%) CSF samples tested positive for SHUV using S segment-specific RT-PCR and confirmed by sequencing. Patients who were positive on the RT-PCR included 85.7% children and 14.3% adults, all with neurological signs, including a 13-day-old infant with hydrops fetalis that had not left the hospital since birth.

Orthobunyavirus genomes are composed of 3 segments of negative-sense single-stranded RNA ((-) ssRNA) designated as large (L), medium (M), and small (S). Most human diagnostic surveillance studies for orthobunyaviruses are conducted by molecular testing such as real-time reverse transcription PCR (RT-PCR) specific to the S segment [1,34–36], however, due to the short viremic phase some cases may likely be missed. For this reason, serological diagnostic assays to detect IgM antibodies are required to identify acute cases that are outside the viremic phase. Various assays have been described for serological detection of viruses within the Simbu serogroups. ELISAs are the most common serological assays for screening large-scale samples. The N-terminal of the Gc protein on the M segment is highly immunogenic and is the major target for neutralising antibodies to orthobunyaviruses [37–39]. Antibodies against selected Simbu serogroup viruses, including SBV, Akabane virus (AKAV), and SHUV, could be differentiated using the Gc-based triplex ELISA in animals [40]. There are a few serological surveillance studies described for orthobunyaviruses in humans [41–44]. Van Eeden et al. conducted a serosurvey using the VNT assay in veterinarians in South Africa in 2011–2012 and described a 3.9% prevalence of antibodies against SHUV in this group [45].

Currently, there is no commercial ELISA available for SHUV. The development of an IgM ELISA will assist in identifying cases that passed the viremic phase, while an IgG ELISA will assist in determining the seroprevalence on the continent.

The goal of this study was 1.) to establish IgM and IgG serological assays for SHUV for humans and 2.) to implement these together with molecular assays to investigate the incidence of SHUV in cases of acute fever of unknown cause with or without neurological signs (AFDUC/N) in sentinel hospitals in Gauteng and Mpumalanga provinces, South Africa (2019–2021), and 3.) determine the seroprevalence in healthy control patients in the same area.

## Methods

### Ethical statement

Ethical clearance for the study was obtained through the University of Pretoria’s Faculty of Health Sciences ethics committee (100/2017 and 101/2017). Patients were enrolled following written informed consent from patients over 18 and legal guardians of minors and, assent from minors over 12, obtained by surveillance officers who collected demographic and clinical information using case investigation forms and captured it in an anonymous database.

### Sample collection and sample processing

EDTA blood, CSF, and serum specimens from hospitalized patients from Gauteng (Kalafong Hospital) and Mpumalanga (Mapulaneng Hospital and Matikwane Hospital) that displayed acute febrile disease of unknown origin with or without neurological signs (AFDUC/N) were collected through the African Network for improved Diagnostics, Epidemiology and Management of Common Infectious Agents (ANDEMIA) study that ran from January 2019 to December 2021 [46]. Samples were collected throughout the year from all age groups. A total of 992 AFDUC/N cases and 412 controls were enrolled within the study period; the majority of cases were from Gauteng (68.3%), while controls were evenly distributed between sites and obtained from patients hospitalized for other reasons (but without febrile, neurological, respiratory, or enteric infections). Ethical clearance for the study was obtained through the University of Pretoria’s Faculty of Health Sciences ethics committee (100/2017 and 101/2017). Patients were enrolled following written informed consent from patients over 18 and legal guardians of minors and, assent from minors over 12, obtained by surveillance officers who collected demographic and clinical information using case investigation forms and captured it in an anonymous database.

For the purpose of the SHUV surveillance study, samples were randomly selected during the arbovirus season only (Jan- June 2019–2021) and included 345 patients in molecular screening and 225 who had sera available for serology. For the control group, we selected 112 patients randomly during the arbovirus season (Jan -June 2019–2021).

For molecular surveillance, RNA was extracted from CSF and EDTA blood using the Qiagen RNA Viral mini kit (Qiagen, Valencia, USA https://www.qiagen.com). For serological surveillance, either serum or plasma was used. Plasma was separated from red blood cells and buffy coat in EDTA blood samples by centrifugation at 2000 × g for 15 min.

### Differential diagnostics

All AFDUC/N patients enrolled in the ANDEMIA study underwent a standardised syndromic diagnostic workup as part of the broader surveillance protocol [46]. Patients were screened for 32 common and zoonotic pathogens using a macroarray fever/meningitis chip as described in [47] and/or thick and thin blood film microscopy in patients from malaria-endemic Mpumalanga province as part of their standard care. The findings of the ANDEMIA network including South Africa and partner countries was published in Belarbi et al 2025 [48]. Arboviral co-infections were assessed concurrently under the ANDEMIA protocol and included screening for Flavivirus viruses, Alphavirus using RT-PCR and/or IgM serology [49,50]. Differential diagnostic data for cases that tested positive for SHUNV by RT-PCR and/or IgM serology in this study was also analysed for pathogens tested as part of the ANDEMIA study.

### Orthobunyavirus Simbu serogroup specific TaqMan Real-Time (RT-PCR) assay

Although RT-PCR is highly specific for SHUV detection, the characteristically short viremic phase of Simbu serogroup orthobunyaviruses significantly limits the sensitivity of molecular diagnostics in detecting infections that have progressed to symptomatic disease. This diagnostic limitation necessitated the development of complementary serological assays to capture infections sampled outside the acute viremic window.

The Orthobunyavirus Simbu serogroup-specific TaqMan RT-PCR specific to the S gene was carried out using the AgPath-ID One-Step RT-PCR Kit (Thermo Fisher Scientific, Waltham, MA, USA) as described before [4]. RT-qPCR amplicons of 155 base pairs (bp) were used in Sanger sequencing to confirm that positive cases were SHUV. The identity of positive amplicons were initially determined using the Basic Local Alignment Search Tool (BLAST) (https://blast.ncbi.nlm.nih.gov/Blast.cgi). Phylogenetic analysis was performed following multiple sequence alignments, using MAFFT software (https://mafft.cbrc.jp/alignment/server/index.html) with representative strains from the Simbu serogroup and orthobunyavirus genus and edited using BioEdit sequence alignment editor after trimming off the primer regions (v7.0.5.3 software). Maximum-likelihood analysis of 1000 bootstrap repetitions was carried out to construct a phylogenetic tree using MEGA X (http://www.megasoftware.net) as confirmation that the amplicons represent SHUV.

Viral Load Quantification: Cycle threshold (CT) values were recorded for all positive RT-qPCR samples, although absolute quantification of viral genome copy numbers per ml was not performed. CT values provide semi-quantitative information, with higher CT values [28–33] detected for RTPCR positive cases detected in this study, indicating comparatively low viral RNA abundance and detection during the waning phase of viremia. Limited sample volume and low RNA levels did not allow more in-depth sequence analysis. Since the sequences were too short to submit to genbank it is supplied in the supplementary files.

### Viral neutralization assay (VNT)

Sera/plasma were heat-inactivated at 56°C for 30 min on a heating block in a 1:8 dilution in serum-free cell culture media supplemented with antibiotics. Subsequent dilutions (1:16 and 1:32) were made from the heat-inactivated 1:8 dilutions. A volume of 100 μl sera/plasma dilutions, along with titrated SHUV (using strain SAE18/09 previously isolated from a horse in South Africa), was added to the wells. The plates were incubated at 37°C for an hour in a humidity chamber. Vero cells diluted to 8 x 10^5^ cells/ml in MEM containing 10% Fetal Bovine Serum (FBS) were added to all the wells on the plates. A negative control that didn’t contain serum and a positive control that contained a Shuni1bAn 1070 (mouse hyperimmune ascitic fluid) MHIAF serum (obtained from the Centers for Disease Control and Prevention (CDC), Fort Collins reference collection) were included. The plates were incubated overnight at 37°C, 5% CO_2_ (18–20 hours). Titers were expressed as the reciprocal of the serum dilution that inhibited ≥75% of CPE. A serum sample was considered positive when there was no cytopathic effect (CPE) presented on dilutions ≥1:16.

### SHUV Gc head antigen production

DNA fragments encoding the N-terminal 234 amino acids of the SHUV Gc protein flanked by an N-terminal GP64 signal sequence and a C-terminal twin Strep-Tag were synthesized by GenScript (Piscataway, NJ, USA). Following the cloning of the fragment in a pBAC-3 baculovirus vector, recombinant baculoviruses were generated with the flashBAC ULTRA baculovirus expression system (Oxford Expression Technologies, Oxford, UK). Briefly, transfection mixtures containing the pBAC-3 vector, a bacmid, and Cellfectin II were added to 24-well plates containing 200,000 SF9ET cells per well. Rescued viruses were subsequently amplified in SF9-ET suspension cultures infected at low MOI. For protein production, High Five cells were infected at high MOI according to the manufacturer’s protocol (Thermo Fisher Scientific, Breda, The Netherlands). Proteins were purified from supernatants using Strep-Tactin resin (IBA, Göttingen, Germany) according to the manufacturer’s instructions. Buffers were exchanged to Tris-buffered saline + 200 mM NaCl using Amicon Ultra centrifugal filters (Merck-Millipore, Amsterdam, The Netherlands).

### SHUV human IgM ELISA set-up

The antigens and antibodies were evaluated by checkerboard titrations to optimize the concentrations. A 96 well microtiter plate (Plastpro Scientific (PTY) LTD, Johannesburg, South Africa) was coated with 100 µl of SHUV Gc antigen at a 2 µg/ml concentration, diluted in phosphate-buffered saline (PBS) (ThermoScientific, South Africa), and incubated overnight at 4°C. The plate was washed six times with 300 µl wash buffer containing PBS with 0.1% Tween 20 using the automatic plate washer (Vacutec, Thermo Fisher Scientific). The plate was blocked with 10% low-fat skimmed milk (SPAR, South Africa) for an hour at 37°C. The plate was washed six times with 300 µl wash buffer. A volume of 100 µl test serum was added to a dilution of 1:100 in 2% skimmed milk in duplicates, and the plate was incubated for an hour at 37°C. A negative control serum was included. The plate was washed six times with 300 µl wash buffer (PBS-Tween20). Goat anti-human IgM antibody (SeraCare, South Africa), 0.1 µg/ml, in 2% skim milk with a final volume of 100 µl was added to the plate and incubated for an hour at 37°C. The plate was washed six times with 300 µl 0.1% wash buffer. For detection, mouse anti-goat IgG HRP-conjugated (Merck, South Africa) 1:1000 dilution in 2% skim milk with a final volume of 100 µl was added to the plate. The plate was again washed six times with 300 µl wash buffer. A volume of 100 µl of (2,2′-azino-di-(3-ethylbenzthiazoline sulfonic acid)) ABTS (BioFX, SurModics, Minnesota, US) was added to each well and incubated in a dark chamber for 30 minutes. The reaction was inactivated with 100 µl 1% sodium dodecyl sulphate (SDS). The plate was read at a wavelength of 405 nm using a plate reader (Multiskan FC, Thermo Scientific). The samples that showed to be neutralising against SHUV were compared with samples that didn’t show to be neutralising against SHUV. A cut-off value was determined using the average of VNT-negative sera over a low negative serum plus the standard deviations as described in the statistical analysis.

### SHUV human IgG ELISA set-up

The IgG ELISA was performed similarly to the IgM ELISA except that Goat Anti-Human HRP-conjugated (Merck, South Africa) (1:1000) in 2% Skim milk with a final volume of 100 μl was added.

### Statistical analysis

Descriptive statistics of the patient’s demographics data and odds ratios for common variables and p-values were calculated on Epi-Info version 7.2.0.1 (https://www.cdc.gov/epiinfo/index.html). A cut-off value for a negative test was calculated using the ratio of the average VNT negative tests over the serum negative control (ZRUA1497/21 or ZRUA1486/2) plus 1, 2, or 3 standard deviations as determined by the ROC analysis below.

To assess the overall diagnostic performance of a test, receiver operating characteristic (ROC) curves were carried out using the MedCalc program (https://www.medcalc.org, version 23.1.7). In addition, the MedCal program also calculates the area under the curve (AUC), which is used to measure the accuracy of diagnostic tests. Each tested serum was carried out in duplicates, and the average OD was divided by the negative serum control (ZRUA1497/21 for IgM and ZRU0A1486 for IgG) to get the ratio values. The cut-off value was calculated by the following formula: Mean of sera that was not neutralising against SHUV (VNT-) + 2 Standard deviation of (VNT).

## Results

### Molecular surveillance for SHUV

The. In total, 356 specimens, either CSF and/or whole blood from 349 hospitalized patients from 2019 to 2021, were randomly selected during the arbovirus season (Jan-Jun) and were tested with the in-house orthobunyavirus one-step real-time RT-PCR to the S gene, which detects the Simbu serogroup orthobunyaviruses [4]. Most samples were from 2019, 156/349 (44.70%), while the least were available from 2021, 88/349 (25.21%), for testing (Table 1).

**Table 1:** SHUV RT-PCR positive cases identified in 2019-2021 from hospitalized patients that displayed acute febrile disease of unknown origin with or without neurological signs.

Categories	2019	2020	2021	Total
No. of samples submitted from Jan to Jun				
	156	105	88	349
No. of SHUV positives				
**Hospital**				
Kalafong (Gauteng)	3 (1.92%)	0(0)	0	3 (0.86%)
Mapulaneng (Mpumalanga)	0	0	0	0
Matikwana (Mpumalanga)	0	0	0	0
**Age**				
0 - 12	3 (1.92%)	0	0	3 (0.86)
13 - 18	0	0	0	0
19 - 59	0	0	0	0
≥60	0	0	0	0
**Total**	**3/156 (1.92%)**	**0/105** **(0%)**	**0/88** **(0.0%)**	**3/349 (0.86%)**

In total, 3/349 (0.86%) patients’ EDTA blood specimens tested positive on the orthobunyavirus one-step real-time RT-PCR with a fragment size of 155 bp on the gel and CT values ranging from 28-33 on real-time analysis. The three cases were identified as SHUV by Sanger sequencing followed by BLAST searches and phylogenetic analysis and grouped with other SHUV cases that were previously identified in South Africa on the ML phylogenetic tree (Fig 1). The SHUNV cases had 94%-98% nucleotide identity to each other SHUV strains identified in horses in South Africa. There were insufficient samples left to attempt sequencing of larger regions or the full genome by NGS. All 3 PCR-positive cases were children under the age of 3 from Kalafong Hospital in Gauteng. No CSF samples were available from these patients.

**Fig 1:**
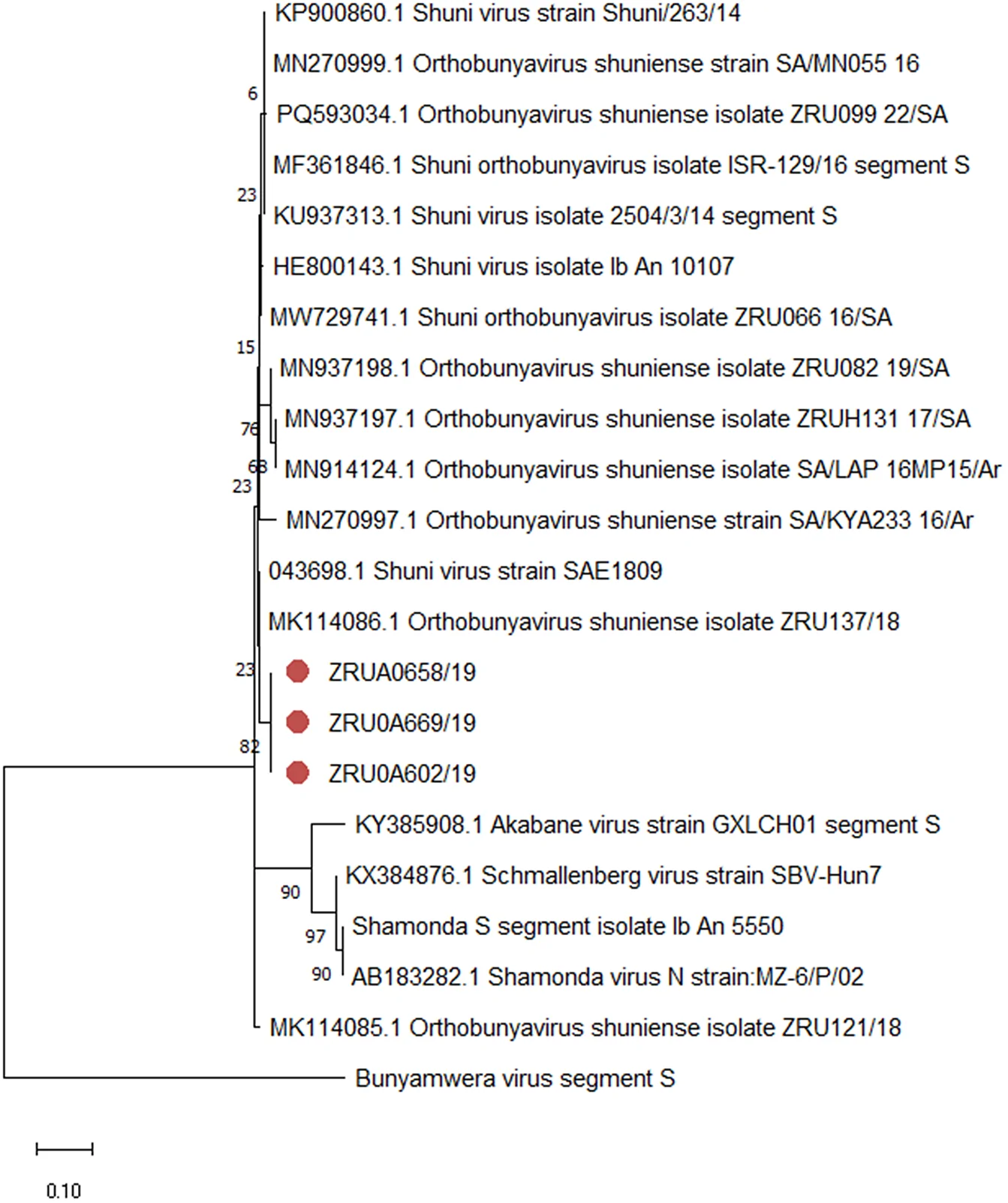
Maximum likelihood phylogenetic tree of positive cases on the orthobunyavirus one-step RT-PCR on the S segment (155 bp) and Sanger sequencing. The phylogenetic analysis of the positive human cases on the S segment from South Africa relative to orthobunyavirus reference strains available on GenBank in the Simbu serogroup. The evolutionary history was inferred by using the maximum likelihood method and the Tamura-Nei model. The percentage of 1000 bootstrap replicates is indicated by the numbers at each node (only values of 50 and higher are shown). Bunyamwera serogroup is included as an outgroup.

### Demographics of patient samples selected for serological assay development

All the patients who had sera or plasma samples available between January-June in 2019–2021 were selected for serological investigations. In total, 225 individual human sera collected in 2019–2021 were screened with the viral neutralization test to identify SHUV seropositive samples. Most of the patients available to screen on the viral neutralization assay were from the age group 0–12 (160/225, 71.11%) and males (126/225, 56.0%). There was an even distribution of sera screened for 2020 (60/225, 26.67%) and 2021 (63/225, 28.00%), with most samples being from the year 2019 (102/225, 45.33%). Most samples were from Gauteng (157/225, 65.78%), followed by Mpumalanga (78/225, 34.67%).

### Viral neutralization test (VNT)

In order to identify SHUV positive sera for use in validation of the IgM and IgG ELISAs all available sera was first screened by VNT assays. In total, we identified 22/225 (9.7%) human sera with neutralising activity against SHUV (Table 2).

**Table 2:** Sera that are positive on the viral neutralization test (VNT) that were tested on PCR, IgM, and IgG ELISA.

Sample ID	VNT	Titer	SHUV PCR	IgM	IgG
**ZRUA0469/19**	Pos	01:16	Neg	Neg	**Pos**
**ZRUA0520/19**	Pos	01:16	Neg	Neg	**Pos**
**ZRUA0521/19**	Pos	01:32	Neg	Pos	**Pos**
**ZRUA0596/19**	Pos	01:16	Neg	Neg	**Pos**
**ZRUA0608/19**	Pos	01:16	Neg	Neg	**Neg**
**ZRUA0628/19**	Pos	01:16	Neg	Neg	**Neg**
**ZRUA1090/20**	Pos	01:16	Neg	Neg	**Neg**
**ZRUA1101/20**	Pos	01:16	Neg	Pos	**Neg**
**ZRUA1121/20**	Pos	01:32	Neg	Pos	**Pos**
**ZRUA1148/20**	Pos	01:32	Neg	Neg	**Pos**
**ZRUA1193/20**	Pos	01:32	Neg	Pos	**Pos**
**ZRUA1230/20**	Pos	01:32	Neg	Pos	**Pos**
**ZRUA1258/20**	Pos	01:16	Neg	Pos	**Neg**
**ZRUA1344/20**	Pos	01:32	Neg	Pos	**Neg**
**ZRUA1349/20**	Pos	01:32	Neg	Pos	**Pos**
**ZRUA1389/20**	Pos	01:16	Neg	Neg	**Neg**
**ZRUA1393/20**	Pos	01:16	Neg	Neg	**Neg**
**ZRUA1489/21**	Pos	01:16	Neg	Pos	**Neg**
**ZRUA1601/21**	Pos	01:32	Neg	Pos	**Pos**
**ZRUA1652/21**	Pos	01:32	Neg	Pos	**Pos**
**ZRUA1712/21**	Pos	01:16	Neg	Neg	**Neg**
**ZRUA1724/21**	Pos	01:16	Neg	Neg	**Neg**
**Total**	22/225 (9.78)		0/22 (0%)	11/22 (50%)	**11/22 (50%)**

### Human SHUV IgM ELISA assay

An IgM ELISA was developed by using positive and negative sera identified by the VNT against SHUV (Fig 2). A total of 22 negative sera were included to determine the cut-off value. To do this, we calculated the mean ratio of VNT negative samples over a serum negative control (Sample ID ZRU0A1497/21 = 1) plus the standard deviations (0.52) x (1,2 or 3) and applied this to the VNT positive and negative serum to determine the most acceptable cut-off value. Table 3 shows the calculation for 1, 2, or 3 standard deviations.

**Table 3:** Estimation of the cut-off value of the IgM and IgG ELISA. Mean (Negative VNT/PBS control) +1, + 2, or 3 Standard Deviations.

a.	Mean + 1 STDV	Mean + 2 STDV	Mean + 3 STDV
IgM cutoff value	1.98	2.50	3.01
AUC	0.89	0.87	0.84
b.			
IgG cutoff value	3.69	4.91	6.1
AUC	0.84	0.83	0.82

(AUC: Area under the curve as calculated in the ROC analysis (section 3.4: STDV: Standard deviation).

**Fig 2:**
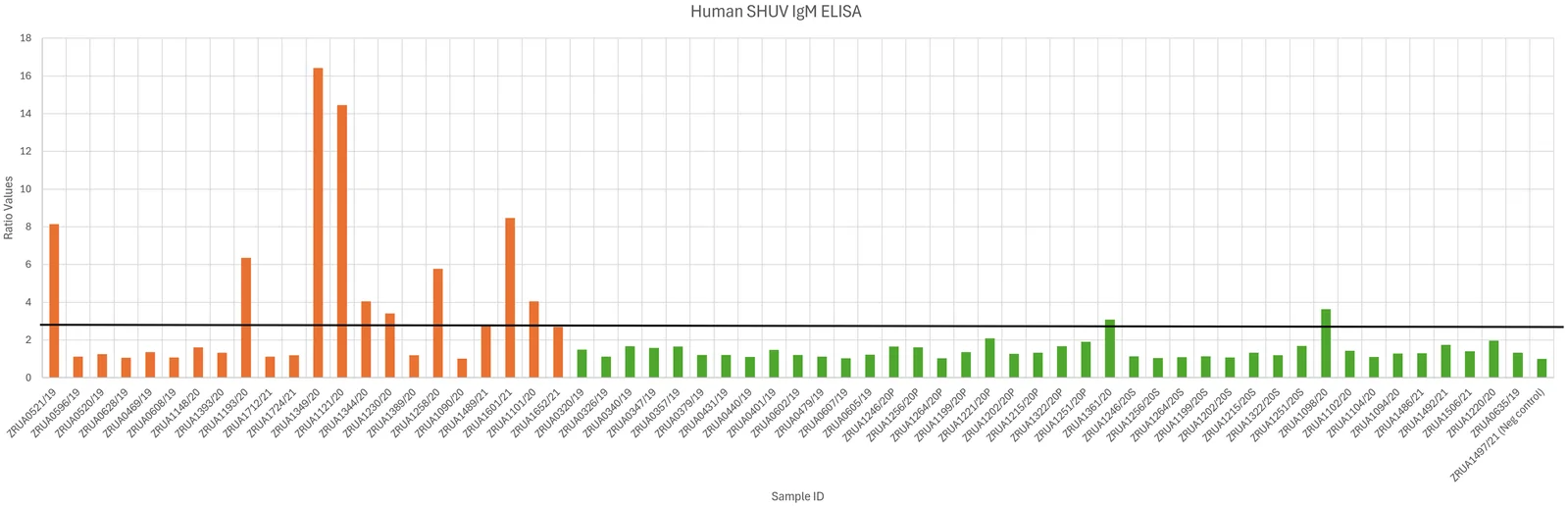
Results of the human IgM ELISA on VNT positive and negative samples. Orange bars show serum that was neutralizing against SHUV and green bars, human serum that was not neutralizing against SHUV from the viral neutralizing assay*.* The black line represents the cut-off vallue.

A few outliers were detected in the negative VNTs that may result in false positives. To ensure the assay remains specific, the mean +2 STDV was selected as the cut-off point for the IgM assay. At 2 standard deviations, the cut-off value was 2.50 (Fig 3). In total, 11/225 (4.89%) human sera tested positive on the IgM assay at this cut-off value. All the IgM-positive sera displayed a titer of 1:16 or 1:32 from the VNT and represented 11/22 (50.00% of VNT positives).

**Fig 3:**
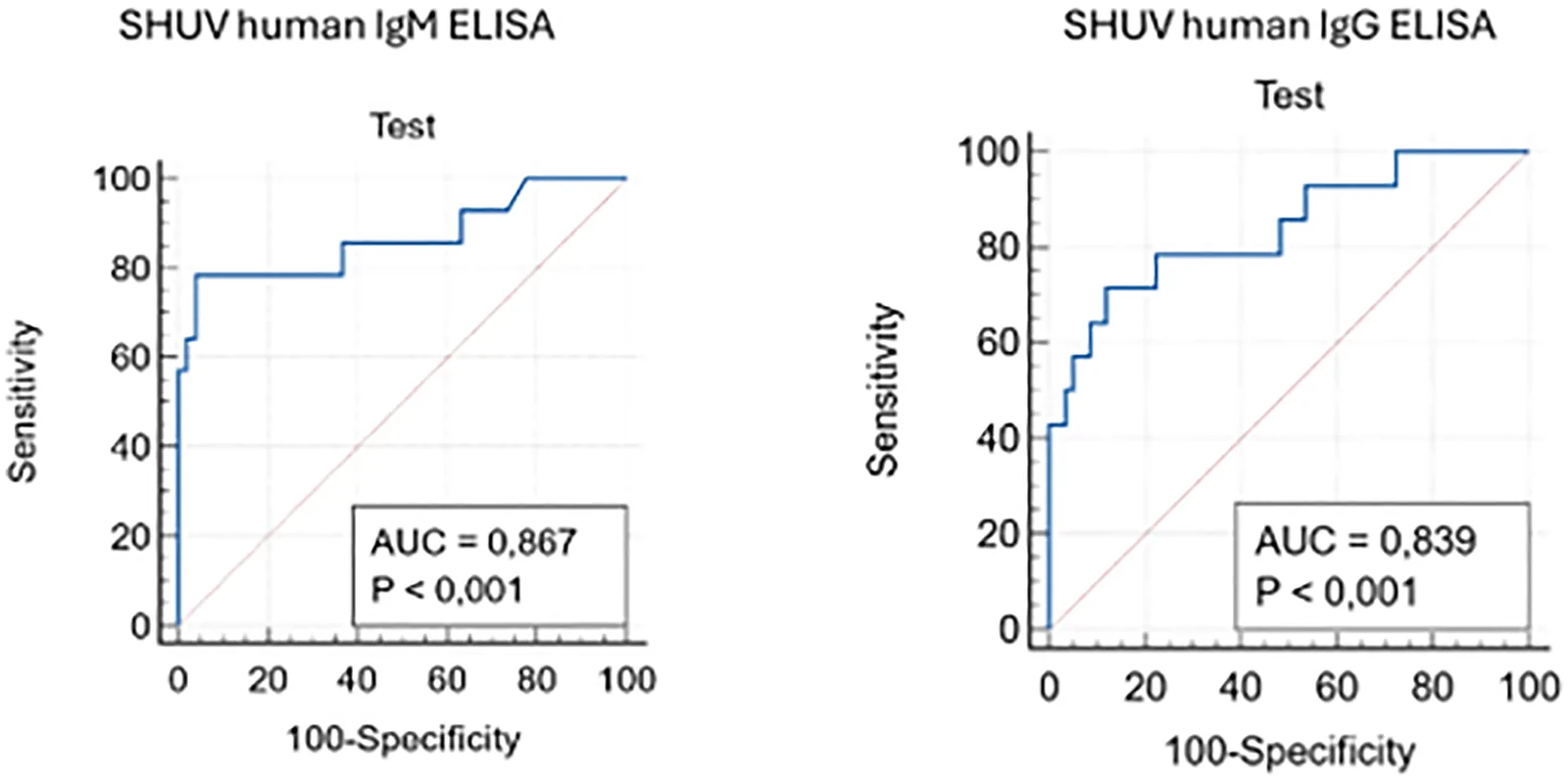
Receiver operating characteristic (ROC) curves of developed SHUV human IgM and IgG ELISA assays from viral neutralization assays of human serum. Area Under the Curve (AUC) was calculated using the MedCal program (https://www.medcalc.org, version 23.1.7).

### Human SHUV IgG ELISA assay

An IgG ELISA was developed by using sera that showed to be neutralising against SHUV from the viral neutralization assay. To do this, we calculated the mean ratio of VNT negative samples over the negative control (Sample ID ZRUA1486/21 = 1) plus the standard deviations (1.21) x (1,2 or 3) and applied this to the VNT positive and negative sera (Table 3).

At 2 standard deviations, the cut-off value was 4.90 using the negative sera, ensuring specificity is higher than sensitivity, thereby reducing false positives (Fig 3).

### Statistical analysis of IgM and IgG ELISA

From the sera that tested positive on the VNT, a total of 11/22 (50,00%) tested positive on the IgM ELISA assay and 11/22 (50.00%) tested positive on the IgG ELISA assay (Table 2). In total, only 7/22 (31.82%) sera tested both IgM and IgG positive, while 7/22 (31.82%) sera didn’t test positive on both the IgM and IgG ELISA despite being VNT positive (Table 2). There were no PCR-positive cases from the VNT sera that were neutralising against SHUV (Table 2).

The efficacy of the two ELISAs was determined by constructing ROC and the AUC was calculated using the MedCal program (https://www.medcalc.org, version 23.1.7) (Fig 3).

The samples used to construct the ROC curves and calculate AUC are indicated in Fig 3 and Appendix 1. The AUC is widely used to measure the accuracy of diagnostic tests and in general, the AUC must be greater than 0.8 to be considered acceptable. The AUC calculated at the selected cut-off values plus 1, 2, or 3 standard deviations were listed in Table 3 to select the most accurate cut off value. From the ROC values, cut-off values plus 2 standard deviations were selected as the most acceptable option to read the IgM and IgG assays where the least false positives would be detected while still sensitive enough to detect true positives. Using 2 standard deviations, the AUC of the IgM ELISA was 0,87 (Fig 3).

The AUC of the IgG ELISA was 0,83 at the cut-off value plus 2 standard deviations which was an acceptable value (Fig 3). This suggests that both the IgM and IgG assay will be acceptable for diagnostic assays where a paired serum is available to measure the rise in IgG. The IgG assay will also be sufficient for serological surveillance in areas without endemic circulation.

### Demographics and risk factors associated with SHUV positive tests of AFDUC/N patients

The demographics per province, per age group, and sex of patients who tested positive for SHUV by VNT vs PCR and IgM, as well as the IgG ELISA is indicated in Table 4.

**Table 4:** Characteristics of SHUV positive samples that were screened by RT-PCR, IgM, and IgG ELISA and VNTs relative to negative human sera indicating the demographics and odds of testing positive.

A: Total positives	No. sample tested: (%)	No. VNT Pos = 22 (%)	No. PCR positives,	No IgM Pos (%)	Combined RT PCR and IgM positive.		
	N = 225	22/225 (9.78%)	3/225 (1.33%)	11/225 (4.89%)	14/225 (6.22%)		
**B: Demographic category**	**No. tested per category:** **n/N(%)**	**No. VNT pos/** **category (%)**	**No. PCR Pos/category (%)**	**No. IgM Pos/category (%)**	**RT PCR & IgM + (%)**	**RT PCR & IgM +** **Odds Ratio (OR)** **(95.00% CI)**	**P-value of combined RT PCR and IgM +**
**Area**							
**Gauteng**	147 (65.33)	14/147 (9.52)	3/147 (2.04)	7/147 (4.76)	10/147 (6.80)	1.35 (0.41-4.45)	0.84
**Mpumalanga**	78 (34.67)	8/78 (10.26)	0/78 (0)	4/78 (5.13)	4/78 (8.97)	0.74 (0.22-2.44)	0.84
**Year**							
**2019**	94 (41.78)	6/94 (6.38)	3/94 (3.19)	1/94 (1.06)	4/94 (4.26)	0.54 (0.16-1.77)	0.89
**2020**	65 (28.89)	11/65 (16.92)	0/65 (0)	7/65 (10.77)	7 (10.77)	2.64 (0.89-7.85)	0.13
**2021**	66 (29.33	5/66 (7.58)	0/66 (0)	3/66 (4.55)	3/66 (4.55)	0.64 (0.17-2.38)	0.71
**Age (years)**							
**0 - 12**	160 (71.11)	12/160 (7.50)	3/160 (1.87)	4/160 (2.50)	7/160 (4.38)	0.38 (0.13-1.13)	0.13
**13 - 18**	3 (1.33)	0/3 (0)	0/3 (0)	0/3 (0)	0/3 (0)	0 (Undefined)	1.00
**19 - 59**	58 (25.78)	9/58 (15.52)	0/58 (0)	6/58 (10.34)	6/58 (10.34)	2.29 (0.76-6.92)	0.23
**≥60**	4 (1.78)	1/4 (25.00)	0/4 (0)	1/4 (25.00)	1/25 (25.00)	5.33 (0.52-54.90)	0.60
**Sex**							
**Female**	99 (44.00)	12/99 (12.12)	1/99 (1.01)	4/99 (4.04)	5/99 (5.05)	0.69 (0.22-2.13)	0.71
**Male**	126 (56.0)	10/126 (7.93)	2/126 (1.59)	7/126 (5.56)	9/126 (7.14)	1.45 (0.47-4.46)	0.71
**Clinical Signs**	Of total tested		In PCR pos	In IgM & VNT pos	In RTPCR or IgM/VNT pos	OR	P-value
**Febrile seizures/ seizures**	32 (14.22)		0/32 (0)	3/32 (9.38)	**3/32 (9.38)**	**1.71 (0.45-6.51)**	**0.69**
**Meningitis**	75 (33.33)		0/75 (0)	6/75 (8.00)	**6/75 (8.00)**	**1.54 (0.52-4.62)**	**0.63**
**Febrile convulsion**	79 (35.11)		3/79 (3.80)	2/79 (2.53)	**5/79 (6.33)**	**1.03 (0.33-3.18)**	**1.00**
**Other**	39 (17.33)		0/39 (0)	0/39 (0)	**0/39 (0)**	**0 (Undefined)**	**0.16**

The highest VNT positivity was detected in the Gauteng province with VNT seroprevalence of 14/147 (9.52%), and in children of the age group of 0–12, 12/160 (7.50%). There was no positive neutralising serum against SHUV in the age group 13–18 (0/3), this could be due to a low number of sera available for testing for this age group. The odds (Odd Ratio, OR) of testing IgM and PCR positive was the greatest in Gauteng, 10/147 (6.80%) (OR=1.35, 95.00% CI (0.41-4.45) and in the year 2020, 7/65 (10.77) (OR= 2.64, 95.00% CI (0.89-7.85)) although not statistically significant (p > 0.05). The highest positivity detected using IgM and PCR was in the age group 18–59, 7/160 (4.38%). However, the odds of testing IgM and PCR positive were the greatest in the age group ≥60, although this may be biased as there were very few samples tested in this age group.

The odds of testing IgM and PCR positive were greatest in males, OR=1.45, 95.00% CI (0.47-4.46), and were statistically significant (p ≤ 0.05). Gauteng province had the highest seroprevalence 7/147 (4.76) and the year 2020 and had the highest percentage of positives 5/65 (7.69), respectively. The IgG ELISA set was used to determine seroprevalence in healthy controls described below.

An overview of the clinical signs and demographics of the SHUV IgM-and PCR-positive patients can be seen in Table 5. All 14 positive patients had neurological signs. All 3 patients who tested positive on PCR had febrile seizures and were all children under the age of 3. In total, 6/14 (42.8%) of those who tested positive on the IgM ELISA assay had meningitis and were between11–43 years of age. A 21 and 82 year old patient 2/14 (14.2%) had seizures. Four (28.5%) of patients with meningitis were HIV + , and between 31–43 years old. One adult HIV negative patient also tested positive for Epstein Barr Virus (EBV) on serum (7.1%).

**Table 5:** Clinical signs in acute SHUV RT-PCR and/or IgM/VNT-positive AFDUC/N patients. The main clinical signs as well as underlying conditions and other pathogens detected through differential diagnostic testing in the ANDEMIA study.

Sample ID	RT-PCR/IgM&VNT+	Year	Age	Sex	Area	Main symptoms	Other Chronic illnesses	Co-infection detected through Fever Chip (RT-PCR)	One month follow-up performed	Clinical outcomes at one-month follow-up
ZRUA1230/20	IgM+	2020	1	M	Gauteng	Febrile seizures, fatigue, weightloss		None	No	
ZRUA0669/19	PCR+	2019	1	M	Gauteng	Febrile convulsions		None	Yes	Recovered
ZRUA0665/19	PCR+	2019	1	F	Gauteng	Febrile convulsions, fatigue		None	Yes	Recovered
ZRUA0521/19	IgM+	2019	2	F	Gauteng	Febrile seizures, fatigue, weight loss		None	No	
ZRUA0658/19	PCR+	2019	2	M	Gauteng	Febrile convulsions, fatigue, weight loss		None	Yes	Recovered
ZRUA1101/20	IgM+	2020	3	F	Gauteng	Febrile convulsion, fatigue, weight loss		None	Yes	Recovered
ZRUA1193/20	IgM+	2020	11	M	Gauteng	Meningitis, headache		EBV	Yes	Recovered
ZRUA1258/20	IgM+	2020	21	M	Mpumalanga	Seizures, headache, fatigue		None	Yes	Not fully recovered, clinical improvement
ZRUA1601/21	IgM+	2021	29	M	Mpumalanga	Meningitis, headache	Suspected Cryptococcal meningitis	None	No	
ZRUA1652/21	IgM+	2021	31	M	Gauteng	Meningitis, headache, fatigue	HIV+	None	No	
ZRUA1349/20	IgM+	2020	38	M	Gauteng	Meningitis, headache, fatigue, stiff neck, blurred vision	HIV+	None	Yes	No clinical improvement
ZRUA1344/20	IgM+	2020	43	F	Mpumalanga	Meningitis, headache, fatigue, chills	HIV+	None	Yes	Recovered
ZRUA1489/21	IgM+	2021	43	F	Gauteng	Meningitis, headache, fatigue	HIV+	None	No	
ZRUA1121/20	IgM+	2020	82	M	Mpumalanga	Seizures		None	No	

One-month follow-up data were available for 8 of 14 participants (57.1%). Of these, six (75.0%) had fully recovered, one (12.5%) demonstrated clinical improvement but had not fully recovered, and one (12.5%) reported no clinical improvement. This patient was also HIV + .

### Demographics of SHUV seroprevalence using IgG ELISA in the healthy control group

The AFDUC/N VNT positive and negative samples were used to establish an IgG ELISA. To do this, we calculated the mean ratio of VNT negative samples over the negative control (Sample ID ZRUA1486/21 = 1) plus the standard deviations (1.21) x (1,2, or 3). At 2 standard deviations, the cut-off value was 4.91.

In total, 9/112 (8%) tested positive. The highest seropositivity was in the age group 0–12, 7/42 (16.67%). This age group was most likely to be exposed to SHUV (OR=6.80, 95,00% CI (1.34 –34.48)) as compared to other age groups and showed to be statistically significant (p ≤ 0.05) (Table 6). Females had the highest sero-positivity, 7/48 (14.58%), as compared to males, 2/64 (3.13%), and females were most likely to be exposed to SHUV (OR=5.29, 95.00% CI (1.05 – 26.75)) but not statistically significant (p > 0.05). The year 2021, 7/75 (9.33%) had the highest seroprevalence. Mpumalanga province, 6/71 (8.45%) had a higher seroprevalence, and residents from this area were most likely to be exposed to SHUV (OR=1.17, 95.00% CI (0.27 – 4.95)), however, not statistically significant (p > 0.05). Positive cases under the age of 12 were mostly children who came to the hospital for their immunization shots, while the two adult sero-positive individuals were hospitalised patients with gunshot wounds.

**Table 6:** Demographics of the seropositive Shuni Virus cases in the Gauteng and Mpumalanga area in the healthy control group.

A: Total positives	No. sample tested: (%)	No IgG/Neut Pos (%)		
	N = 112	9/112 (8.04%)		
**B: Demographic category**	No. tested per category: n/N(%)	No. IgG Pos/category (%)	Odds Ratio (OR) (95.00% CI) IgG	P-value IgG Pos
**Area**				
**Gauteng**	41 (36.61)	3/41 (7.31)	0.86 (0.20 – 3.62)	1.00
**Mpumalanga**	71 (63.39)	6/71 (8.45)	1.17 (0.28 – 4.95)	1.00
**Year**				
**2019**	11 (9.82)	1/11 (9.09)	1.16 (0.14 – 10.27)	1.00
**2020**	26 (23.21)	1/26 (3.85)	0.39 (0.05 – 3.27)	0.63
**2021**	75 (66.96)	7/75 (9.33)	1.80 (0.36 – 9.14)	0.73
**Age (years)**				
**0 - 12**	42 (37.50)	**7/42 (16.67)**	**6.80 (1.34 –34.48)**	**0.025**
**13 – 18**	5 (4.46)	0/5 (0)	0 (undefined)	1.00
**19 – 59**	61 (54.46)	2/61 (3.28)	0.21 (0.042-1.08)	0.10
**≥60**	4 (3.57)	0/4 (0)	0 (undefined)	1.00
**Sex**				
**Female**	48 (42.86)	7/48 (14.58)	5.29 (1.047 – 26.75)	0.06
**Male**	64 (57.14)	2/64 (3.13)	0.19 (0.04 – 0.96)	0.06

## Discussion

Most surveillance studies on SHUV have been conducted in animals with clinical signs ranging from acute febrile illnesses to neurological signs, abortion, and birth defects in Africa and Israel [2–6]. More recently, the first detection of SHUV in humans in South Africa was reported following screening of CSF samples from hospitals in the Gauteng province using molecular test [1]. Most of the hospitalized patients were children who displayed neurological signs of seizures, and other signs of diarrhoea, rash, coughing, otitis media, fever, and difficulty breathing [1]. In addition, one of the cases was a 13-day-old child who had not left the hospital since birth with hydrops fetalis, suggesting likely mother-to-child transmission. Studies in ovine suggested that SHUV transmission may occur from the mother to fetus [51] while other orthobunyaviruses cause abortion or congenital malformations in ruminants and porcine [52,53]. Orthobunyaviruses that cause infection in humans include La Crosse virus, which causes encephalitis in children in the U.S [44,54], and Ngari virus, which is associated with haemorrhagic fever signs in Africa [26–28]. Cache Valley virus, which is associated with meningitis and encephalitis in the USA [34,55]. OROV, which was previously associated with febrile illness in Brazil, has in the past year emerged in the Caribbean, across South America, reaching over 11.000 cases, including 2 deaths [56]. Imported cases in the USA, Canada, and Europe have raised concern about the potential spread of orthobunyaviruses beyond their current geographical range [57]. OROV and SHUV are both transmitted by *Culicoides,* while SHUV is also transmitted by endemic *Aedes* and *Culex* mosquitoes in Africa [8,12,16]. Currently, no data is available about the epidemiology of SHUV in human cases in other African countries or Israel. This is partially due to the lack of sensitive molecular and serological assays.

In this study, we aimed to use molecular and IgM sero-diagnostic assays to determine the incidence of SHUV in patients with AFDUC/neurological cases in hospitals in Gauteng and Mpumalanga in 2019–2021 as well as the seroprevalence in healthy control patients. RT-PCR only identified SHUV RNA in 3/349 (0.8%) cases of a cohort of human patients hospitalized with febrile and neurological signs. All (n = 3) were children under the age of 3 and hospitalised in Kalafong Hospital in Pretoria, Gauteng. All 3 patients were hospitalized due to febrile convulsions, coughing, and seizures were also recorded as clinical signs. RT-PCR was effective in our previous study for detecting SHUV in 3% of CSF samples from human patients at the same hospital in South Africa, with most cases also occurring in children [1]. The addition of an IgM ELISA for serological diagnoses in our current study increased the overall positive rate to 4.8%. Combining the RT-PCR and IgM serology results suggests that 6.22% of AFDUC/N cases were due to SHUV during the arbovirus season. From the IgM ELISA assay, there was a high incidence in adults 19–59, 6/58 (10.34%). Patients presented with clinical signs of either febrile convulsions, 5/14 (35.71%), meningitis, 6/14 (42.86%), or seizures, 3/14 (21.42%). SHUV has previously been associated with meningitis and febrile convulsions in young children and in adults [1]. While 28% of adult patients with meningitis were also HIV positive, and one had a EBV co-infection, most cases described here had SHUV as the only detected pathogen. The patients for who follow up data was available mostly recovered after a month although one HIV positive patients did not.

The low molecular detection rate observed in this study reflects a critical diagnostic challenge inherent to Simbu serogroup viruses: RT-PCR is challenging for diagnosing these viruses due to their characteristically short viremic phase. Typically, when clinical signs are acute, particularly in hospitalized patients, the viremic window may already have passed. By the time a patient develops notable neurological symptoms warranting clinical investigation, virus is already cleared and RNA become extremely difficult to detect, even with highly sensitive molecular assays. This diagnostic limitation supports the findings of our study: the 0.86% RT-PCR positivity rate may significantly underestimate the true burden of SHUV infection in the patient population. The substantially higher serological positivity rate (9.7% by VNT; 4.89% by IgM ELISA) compared to molecular detection suggests many infections were sampled outside the brief viremic window. Therefore, complementary serological assays are essential for comprehensive surveillance and epidemiological investigations of Simbu serogroup viruses.

Gauteng had a higher incidence of SHUV RT-PCR and/or IgM positive cases, with 10/147 (6.80%) patients testing positive and a slightly higher odds of contracting SHUV (OR=1.35, 95.00% CI (0.41-4.45)) than Mpumalanga, although not statistically significant. This may be biased since more cases were submitted from Kalafong Hospital in Gauteng. IgG ELISA was used to determine the seroprevalence in a control group consisting of patients without neurological, respiratory, or enteric signs from the three hospitals (Kalafong Hospital, Mapulaneng Hospital, and Matikwana Hospital). From the results, children under the age of 12 (7/43 (16.2%) had the highest seropositivity of all age groups, suggesting children may be exposed at a young age.

Currently, there are no commercial human SHUV ELISAs available. Adding IgM assays could improve the success of identifying positive cases as part of surveillance for SHUV in humans. Since no positive control sera were available for optimizing the ELISA, we opted for screening patient sera with VTN first to identify seropositive cases that are most likely to be due to SHUV. Orthobunyaviruses in the Simbu serogroup may cross react on IgM and IgG serological assays making VNTs an important tool for confirming positive cases for different viruses. Of the 225 AFDUC/N samples screened, 22% had neutralising antibodies to SHUV. Using the IgM ELISA, 11 of these (50%) or 4.49% of the screened samples had IgM antibodies, and 11/22 (50%) had IgG antibodies at the cut-off plus 2 STDV. This suggests that this strict cut-off value likely underestimates the number of positives in both assays. ROC curve analysis indicates AUC values of greater than 0.8 for both assays, suggesting that they performed well enough to be implemented for diagnostic or surveillance settings. Since it is not known to us which VNT-positive cases contained IgM or IgG beforehand, it is not possible to calculate the sensitivity or specificity with these sera of the individual assays. However, both IgM and IgG ELISAs successfully detected antibodies in VNT-positive cases. High background detected in several VNT-negative samples contributed to the selection of the high cut-off value. This background may be due to nonspecific binding to the baculovirus antigens or the presence of antibodies to related orthobunyaviruses in the population. To overcome this we recommend that these assays are implemented with a protocol that includes VNT confirmation of positive cases to reduce the chance of false positives.

This study has several limitations, and these can be improved by increasing the number of samples to validate the assays. Since it is not possible to tell which of the VNT-positive samples are IgM or IgG positive without the IgM and IgG ELISAs, this is not the ideal method for calculating the sensitivity and specificity of the assay since neither assay should detect all VNT positives, and some samples may have both in. The study group represented hospitalized patients with AFDUC and neurological signs in 2 provinces in South Africa, but was skewed towards children, as these are the most representative patient groups in public sector hospitals in South Africa. Investigations should be expanded to other areas of the continent and include both hospitalized and community patients to define the burden of disease. In addition, another potential limitation of this study is the inability to fully assess serological cross-reactivity with other Simbu serogroup viruses. Cross-reactive antibody responses may occur among closely related Simbu serogroup members, in commercial ELISA assays. Data is needed to determine the extend of cross reactions with virus neutralisation tests (VNT) as well. Consequently, ELISA results alone may not reliably distinguish between SHUV exposure and exposure to antigenically similar Simbu serogroup viruses in regions where multiple members of this group are endemic. Although the ELISA developed here was validated using VNT-characterised sera, a comprehensive cross-reactivity assessment was not feasible due to limited availability of well-characterised reference sera and isolates of other Simbu serogroup viruses. To address this, future studies should evaluate the assay against sera positive for other Simbu serogroup viruses known to circulate in Africa, and VNT’s to closely related Simbu serogroup viruses such as Schmallenberg and Shamonda virus which would strengthen specificity claims and provide empirical data on cross-reactivity under field conditions. VNT should be used to confirm SHUV IgM and IgG ELISA positive findings when used in diagnostic or surveillance studies to rule out cross reactions.

To conclude, the study suggests that SHUV is missed as a cause of neurological infections in children and adults in South Africa. Typical signs in hospitalized patients include febrile convulsions, meningitis, and seizures, but further investigations of cohorts of patients that include more adults, as well as patients with birth defects, should be conducted. SHUV was detected in both subtropical and temperate provinces of South Africa. Surveillance studies should be expanded to the rest of the continent to determine the importance of SHUV in neurological infections and birth defects in other regions of Africa and the emergence of SHUV in new regions.

## Supporting information

S1 Fig Validation of the human IgG ELISA against VNT positive and negative samples. Orange bars show serum that was neutralising against SHUV and green bars, human serum that was not neutralising against SHUV from the viral neutralising assay. The black line represents the cut-off value used in the ELISA.
(TIF)

S1 Data Fasta files of the 3 SHUV RTPCR positive samples. (TXT)

S2 Data P-distance analysis of Shunivirus sequences identified in this study to other Simbu serogroup viruses in Fig 1. The strain name and accession numbers are indicated in the column A and row 1 and the percentage difference in the cells in the matrix. The 3 clinical isolates identified in the current study is highlighted in yellow.
(XLSX)
